# Circadian variations in the occurrence of first‐ever intracerebral hemorrhage from different sources of income: a hospital‐based cross‐sectional study

**DOI:** 10.1186/s12883-021-02163-2

**Published:** 2021-03-31

**Authors:** Yan-Yue Wang, Ning Yan, En-Yuan Wang, Yun-Tao Pu

**Affiliations:** 1 Department of Neurology, University-Town Hospital of Chongqing Medical University, University-Town Middle Road 55, 401331 Chongqing, China; 2 Department of Traditional Chinese Medicine, Chongqing University Three Gorges Hospital, Xincheng Road 165, Wanzhou District, 404000 Chongqing, China

**Keywords:** Intracerebral hemorrhage, Circadian variations, Income sources, Risk factors

## Abstract

**Background:**

The onset time of intracerebral hemorrhage (ICH) may be closely related to the working style and living habits of people, which are determined by different income sources in China. Therefore, the purpose of our study was to investigate the Circadian Variations in the occurrence of ICH from different sources of income.

**Methods:**

This retrospective study enrolled 4,327 patients with first-ever ICH. Based on the time of day at which the patients developed symptoms, the classifiable onset time was assigned to one of eight three-hour intervals. And based on different income sources, they were categorized into three groups: Farmers, Wage-earners, and Freelancers. Demographic and risk factors of patients were then summarized, and the circadian variation of the 3 groups of patients’ known time of onset and those stratified by sex and age were analyzed.

**Results:**

The frequency of ICH onset exhibited significant circadian variation among the 3 income groups, demonstrating a bimodal distribution in the daytime, with a nadir during the night (all *P <* 0.001). Three groups showed a significant initial peak between 06:01 and 09:00, and the same peak was observed in their subgroups of sex and age. In the 3 income source groups, there was a smaller second peak that between 15:01 and 18:00 for Farmers and Wage-earners and 18:01 and 21:00 for Freelancers. After stratification by sex and age, the second peak was between 18:01 and 21:00 for female in Farmers, female in Freelancers, under 65 years of age in Wage-earners and 65 years or older in Freelancers, while 15:01 and 18:00 for the other groups.

**Conclusions:**

Different circadian variations of ICH onset time are found in patients with different income sources in southwest China’s Chongqing Municipality cohort. Moreover, the frequency and distribution pattern of peak hours may be closely related to the working style and living habits of people with different income sources.

## Background

Primary intracerebral hemorrhage (ICH) is a common, devastating disease that lacks an effective treatment and is associated with a high rate of death or disability [1–4]. Evidence from epidemiological studies on worldwide stroke victims have indicated that the overall incidence of ICH had been roughly stable at 24.6 per 100,000 person-years, yet the rate has increased to 51.8 in Asian populations [3]. Moreover, the National Epidemiology Survey of Stroke in China (NESS-China) reported that 66.2 out of every 100,000 people suffered from ICH in 2012 to 2013, and the greatest stroke burden was observed in rural areas of northern regions [5].

For nearly 30 years, circadian variation of ICH has been widely investigated around the world [6–22] (Table 1). Nevertheless, the findings in many community- and hospital-based studies were variable or even contradictory. Numerous studies have demonstrated that ICH mostly first occurred in the morning hours [6–14, 20, 21], and some of which were accompanied by a second peak [6–9, 20]. Several researchers have also found the peak occurs between morning and afternoon [17, 22] or after awakening [18, 19]. Furthermore, two additional studies with 527 patients in Asia reported that a single peak was observed in the afternoon hours [15, 16]. In these studies, circadian variation of ICH incidence may be dependent on the methodology used. They divided a day into 2-[6–9, 15, 21], 3-[20], 4-[11], 6-[10, 12–14, 16], 8-hour intervals [17, 22] or more [18, 19]. The different findings concerning the time intervals of statistically significant ICH incidence were identified as: a morning peak (06:00–08:00 h [6], 08:00–10:00 h [7,8], 08:00–12:00 h [11], 10:00–12:00 h [9], 06:00–12:00 h [10,12-14]), sometimes with a second peak (16:00–18:00 h [7] and 18:00–20:00 h [6, 8-9]), or a single peak during the afternoon (18:00–20:00 h [15], 12:00–18:00 [16]), or a single peak from morning to afternoon (08:00–16:00 h [17]), or a peak after awakening [18, 19]. Not only the methodology, the exact peak circadian variation of ICH incidence may also be dependent on sex, age, and living habits [15, 20].

**Table 1:** Available evidence on patterns of onset of ICH for nearly 30 years

Ref.	Author, year	Country	Cases (n)	Peak
[7]^a^	Omama et al., 2006	Japan	3852	06:00-08:00h and 18:00-20:00h
[8]^a^	Spengos et al., 2003	Greece	200	08:00-10:00h and 16:00-18:00h
[9]	Inagawa et al., 2000	Japan	267	08:00-10:00h and 18:00-20:00h
[10]	Sloan et al., 1992	USA	237	10:00-12:00h and 18:00-20:00h
[11]	Fodor et al., 2014	Romania	94	06:00-12:00h
[12]^a^	Butt et al., 2009	Pakistan	329	08:00-12:00h
[13]	Passero et al., 2000	Italy	778	06:00-12:00h
[14]	Gallerani et al., 1994	Italy	161	06:00-12:00h
[15]	Ricci et al., 1992	Italy	375	06:00-12:00h
[16]^a^	Inagawaet et al., 2003	Japan	350	18:00-20:00h
[17]	Cheung et al., 2001	China	177	12:00-18:00h
[18]^a^	Nyguist et al., 2001	USA	137	08:00-16:00h
[19]^a^	Turin et al., 2013	Japan	335	Awake
[20]	Nagakane et al., 2006	Japan	129	Awake
[21]	Kocer et al., 2005	Turkey	240	03:00-06:00h and 15:00-18:00h^b^
[22]	Wroe et al., 1992	UK	66	10:00-12:00h^b^
[23]^a^	Kelly-Haues et al., 1995	USA	43	08:00-16:00h^b^

^a^The subjects were first-ever ICH, ^b^Peak had no significant

China is a country with a large agricultural industry, the population of farmers numbers nearly 600 million. Chongqing is a province located in the southwest of China, and the two centers involved in this study, University-Town Hospital of Chongqing Medical University and Chongqing University Three Gorges Hospital, are located in the west and east of Chongqing respectively. A report from the National Bureau of Statistics shows that as of 2019, Chongqing has a permanent population of more than 30 million, of which non-urban residents account for about 40 %. The composition of Chongqing’s labor force is still dominated by the primary industry such as planting, forestry and animal husbandry, which accounts for more than 40 %, followed by the secondary industry dominated by processing and manufacturing, which accounts for about 20 %, and finally the tertiary industry dominated by commerce and service industry, which accounts for more than 30 %. Farmers’ income mainly comes from agricultural production, while for urban population, their main sources of income are wages and non-fixed income. People with different income sources differ in working style and living habits, and these factors may be related to the circadian variations in the occurrence of ICH.

Therefore, our aim was to investigate circadian variations in the occurrence of ICH from different sources of income, as well as observe whether the onset time of ICH was affected by the working style and living habits.

## Methods

### Study population

 This cross-sectional study was approved by the ethics committee of University-Town Hospital of Chongqing Medical University. We screened 4,327 patients with ICH from databases of University-Town Hospital of Chongqing Medical University and Chongqing University Three Gorges Hospital (including Neurological Department, Neurosurgery, First aid Branch, Baian Branch and Yuan Branch) from January 1, 2012 to December 31, 2017. All patients underwent complete cranial computed tomography (CT) scans and/or magnetic resonance imaging (MRI) within 24 h after onset of symptoms. The diagnosis of ICH was confirmed by local neurologists and/or neurosurgeons based on the standards of the literature [23]. We exclude cases involving traumatic ICH, subarachnoid hemorrhages (SAH), those with a history of ICH, and case where exact hours or approximate time intervals of onset could not be determined. All the variables including sex, age, occupation, income source, date and hour of onset, situation at onset, health-related behaviors, and past medical history were recorded. We divided the patients into three groups based on the different income sources: i.e. Farmers (whose income mainly came from agricultural production), Wage-earners (whose income mainly came from wages) and Freelancers (whose income were unfixed).

### Data analysis

Based on the data provided by the medical records, the classifiable onset time was assigned to one of eight three-hour intervals: 00:01–03:00; 03:01–06:00; 06:01–09:00; 09:01–12:00; 12:01–15:00; 15:01–18:00; 18:01–21:00; 21:01–24:00 h. Distributions of ICH onset in the 8 onset-time partitions were analyzed in the overall population, in the 3 income source groups and subgroups of income sources further stratified by sex and age. To examine the effect of age on the time of onset, the patients were stratified into *<* 65 and ≥ 65 years. The risk factors of ICH in different income source groups were assessed as follows. Hypertension(HTN)was defined as those with an average (calculated from three measurements) systolic blood pressure (SBP) ≥ 140 mmHg and/or an average diastolic blood pressure (DBP) ≥ 90 mmHg, a history of hypertension, or prior use of antihypertensive agents. However, cases of prehypertension (PHT, representing a SBP of 120–139 mmHg and/or a DBP of 80–89 mmHg) were not included [24]. Dyslipidemia was defined as to be those with total cholesterol (TC) ≥ 5.2 mmol/L, triglyceride (TG) ≥ 1.7 mmol/L, low-density lipoprotein cholesterol (LDL-C) ≥ 3.4 mmol/L [25], or the current use of anti - lipidemic medication. Diabetes mellitus (DM) was defined to be the cases with a history of DM or fasting blood glucose (FPG) level ≥ 7.0 mmol/L [26]. The variable Alcohol Consumption is defined to be those that self-reported consuming alcohol more than three times per week. Smoking was defined as current regular use (any amount).

### Statistical analyses

All analyses were carried out with SPSS version 24.0 software (IBM Inc., Amonk, NY). Mean ages were expressed as mean (SD), while other variables including demographics and risk factors, were presented as values or percentages. Analysis of variance was used for mean age comparison among the 3 income source groups, and demographics and risk factors were compared by χ^2^ test. χ^2^ test was used to compare the distribution of ICH by 3-h time intervals among them. In addition, to screen out confounders, stratified analyses were used to assess the contribution of demographic and risk factors to the temporal distributions of ICH in the overall population. Variables used were age, sex, hypertension, dyslipidemia, diabetes mellitus, alcohol consumption, and smoking. Assuming that time of day has no effect on the onset of ICH, we should observe a roughly uniform distribution. Data were statistically analyzed with χ^2^ test for goodness of fit to the model of equal distribution of ICH to evaluate the circadian variations in ICH onset. To estimate the relative risks (RR) with 95 % confidence intervals (CI) of ICH occurring at specific time periods respectively, the observed number of ICH was compared with the average number of 8 3-h intervals. RR adjusted for confounders by cox regression analysis. *P <* 0.05 was considered significant.

## Results

### Patient characteristics

4,150 patients with known onset time were recruited in our study. Among them, 2,126 were Farmers, 1,194 were Wage-earners and 830 were Freelancers. The remaining 177 patients with no identifiable times of onset were excluded from further analysis. Table 2 shows the demographics and risk factors of all patients afflicted with their first ICH in Farmers, Wage-earners, and Freelancers. There were significant differences in demographics and risk factors except dyslipidemia among the 3 income source groups (all *P <* 0.05). There were also significant differences in the temporal distributions of ICH among the 3 income source groups. After stratified by the demographics and risk factors in the overall population, the differences in the temporal distributions of ICH were observed in age, sex, and hypertension strata (all *P <* 0.05), whereas there were no significant differences in the other risk factor strata.

**Table 2:** Demographics and risk factors of the patients with ICH

	Farmers (*n*=2126)	Wage-earners (*n*=1194)	Freelancers (*n*=830)	Total (*n*=4150)	*P *
Age, years, mean (SD)	62.9 (11.0)	63.2 (13.0)	59.8 (13.5)	62.4 (12.2)	<0.001^*^
Age^a^, n (%)					
<65	1107 (52.1)	594 (49.7)	520 (62.7)	2221 (53.5)	<0.001
≥65	1019 (47.9)	600 (50.3)	310 (37.3)	1929 (46.5)	
Sex^a^, n (%)					
Male	1152 (54.2)	860 (72.0)	519 (62.5)	2531 (61.0)	<0.001
Female	974 (45.8)	334 (28.0)	311 (37.5)	1619 (39.0)	
Risk factors, n (%)					
Hypertension^a^	1863 (87.6)	1047 (87.7)	700 (84.3)	3610 (87.0)	0.040
Dyslipidemia	710 (33.4)	440 (36.9)	307 (37.0)	1457 (35.1)	0.060
Diabetes mellitus	88 (4.1)	112 (9.4)	58 (7.0)	258 (6.2)	<0.001
Alcohol consumption	489 (23.0)	371 (31.1)	201 (24.2)	1061 (25.6)	<0.001
Smoking	551 (25.9)	368 (30.8)	241 (29.0)	1160 (28.0)	0.008

*SD *Standard deviation. *P *for the differences in demographics and risk factors among Farmers, Wage-earners and Freelancers*. *Variables used were age, sex, hypertension, dyslipidemia, diabetes mellitus, alcohol consumption, and smoking. *P *values are based on χ^2^ test if not indicated otherwise. ^*^P value based on variance analysis. ^a^Denotes confounders screened out by stratified analyses in demographic and risk factors.

### Income sources and circadian variations

**Fig. 1:**
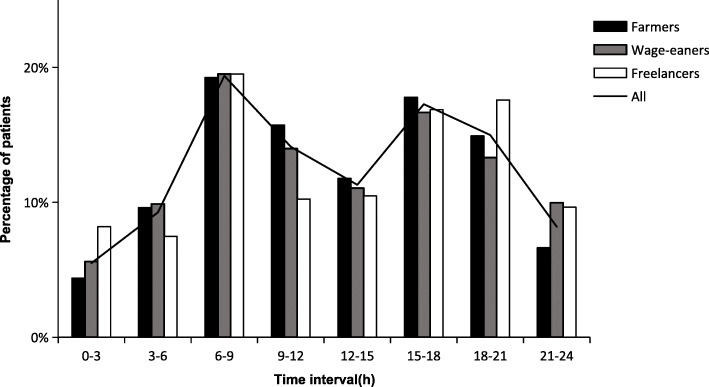
Time-specific onset percentage in patients with ICH stratified by sources of income

**Fig. 2:**
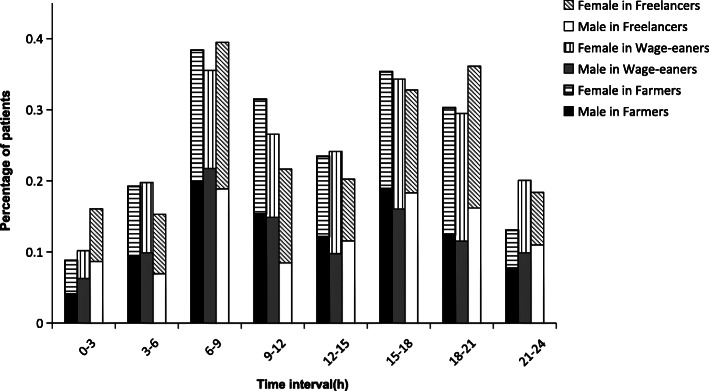
Time-specific onset percentage in patients with ICH stratified by sex

**Fig. 3:**
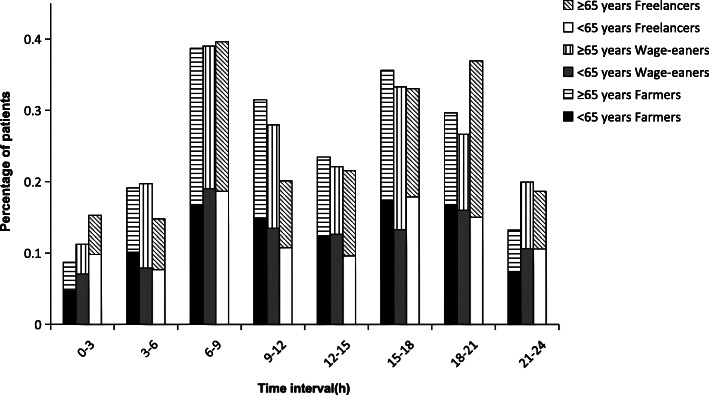
Time-specific onset percentage in patients with ICH stratified by age

In the 3 income groups and their subgroups stratified by age and sex, the circadian variations of onset time were bimodal in all cases; there was one significant initial peak during the period of 06:01–09:00, a smaller second peak during 15:01–21:00, and a nadir during the night (all *P <* 0.001). The smaller second peak for each group, however, was different; In the 3 income source groups, the smaller second peak was 15:01–18:00 for Farmers and Wage-earners, and 18:01–21:00 for Freelancers (Fig. 1). In the sex subgroups, all showed a second peak between 15:01 and 18:00 except female in Farmers and female in Freelancers, whose second peak was between 18:01 and 21:00 (Fig. 2). In the age subgroups, the second peak was 18:01–21:00 for the group under 65 years of age in Wage-earners and 65 years or older in Freelancers, while 15:01 and 18:00 for the other groups (Fig. 3). RR values for each peak period were statistically significant after adjustment for age, sex, and hypertension (Table 3).

**Table 3:** The peak time of ICH and relative risk values

	Farmers		Wage-earners		Freelancers	
	Peak time (h)	RR^a^(95%CI)	Peak time (h)	RR^a^ (95%CI)	Peak time (h)	RR^a^ (95%CI)
Total						
	6-9	1.63 (1.37-1.93)	6-9	1.72 (1.37-2.15)	6-9	1.49 (1.14-1.96)
	15-18	1.50 (1.26-1.78)	15-18	1.23 (1.02-1.58)	18-21	1.29 (1.03-1.61)
Sex						
Male	6-9	1.68 (1.34-2.12)	6-9	1.86 (1.41-2.44)	6-9	1.52 (1.09-2.13)
	15-18	1.51 (1.20-1.91)	15-18	1.35 (1.04-1.77)	15-18	1.44 (1.03-2.02)
Female	6-9	1.51 (1.17-1.95)	6-9	1.69 (1.09-2.62)	6-9	1.53 (1.03-2.30)
	18-21	1.37 (1.06-1.76)	15-18	1.42 (1.01-2.01)	18-21	1.56 (1.01-2.41)
Age						
<65years	6-9	1.43 (1.12-1.81)	6-9	1.55 (1.13-2.13)	6-9	1.42 (1.01-1.99)
	15-18	1.43 (1.12-1.82)	18-21	1.50 (1.08-2.09)	15-18	1.44 (1.06-1.97)
≥65years	6-9	1.87 (1.47-2.39)	6-9	1.94 (1.41-2.68)	6-9	1.59 (1.02-2.48)
	15-18	1.57 (1.23-2.01)	15-18	1.58 (1.14-2.19)	18-21	1.73 (1.12-2.68)

*RR *indicates relative risk, *CI *indicates confidential interval. ^a^*RR *values were the observed number of ICH compared with the average number of 8 3-h Intervals by χ^2^ test, and adjusted for sex, age, and hypertension by cox regression analysis.

## Discussion

We investigated the circadian patterns of more than 4,000 patients with ICH from different income sources. To our knowledge, this is the largest study of circadian variation of onset time of ICH available in the literature so far. Our research found there were different circadian variations of ICH onset time in patients with different income sources, that may be related to their different working style and living habits.

The present study showed a significant morning peak between 06:01 and 09:00 in 3 different income source groups as well as in those subgroups further stratified by sex and age. This early morning peak was similar to the finding by Omama et al. [6] and consistent with the striking rise of blood pressure (BP) for 2 to 3 h after awakening [27]. The aforementioned papers observed that it is more common that ICH attacks in the morning hours than any other time of the day. They attribute this to a complex interaction of many factors, with the result that a sudden increase in BP emerging as an explanation for this pattern of onset [6–14, 19–22]. Metoki et al. [28] found that the risk of hemorrhagic stroke would be increased only when the morning mean SBP soared by more than one-fifth (≥ 25 mmHg), regardless of nocturnal blood pressure patterns. The risk of ICH was also exacerbated by extreme drops in SBP (≥ 20 mmHg nocturnal decline in SBP from the diurnal level). Some authors have comprehensively analyzed the endogenous and exogenous factors which influence morning BP surge [29, 30]. A report studied by Morris et al. that observed 14 healthy, sedentary, nonsmoking, nonobese, and normotensive male (aged 19–50 years) also indicated that the morning BP increase was attenuated during bed rest, suggesting that the adoption of an upright posture and/or physical activity in the morning contributes to the morning BP surge [31]. Furthermore, racial differences may also affect morning BP surge [32]. In the existing literature, alcohol intake has been identified as a significant risk factor for ICH. It was reported that people who had heavy alcohol intake (alcohol intake ≥ 46 g/d) had higher BP in the morning and increased the risk of stroke, especially hemorrhagic stroke [33, 34]. In our findings, we observed that the Wage-Earners group had the highest proportions of heavy drinkers, smokers, dyslipidemia, and DM, but the morning peak was in accord with the others. It seems that none of the common risk factors correlates alone with the morning BP surge of ICH occurrence but it may have a closer tie with physical and/or emotional activity after waking.

Research revealed that nearly 40 % of Chinese residents tend to take a nap during the afternoon [35]. Spengos et al. considered the close relationship between the late afternoon peak of onset of ICH and afternoon sleep, the siesta [7]. Wage-earners and Farmers have relatively fixed work schedules, and most of them also have a habit of taking a siesta. This may account for the pronounced afternoon peak. On the contrary, Freelancers usually didn’t have a fixed schedule and the habit of taking a siesta due to their irregular working hours, that could explain why they didn’t display a same peak distribution pattern in the afternoon as Wage-earners and Farmers.

When analyses were performed by dividing the patients according to sex and age, there were partial differences in the afternoon peak, which was consistent with the findings of Inagawa T et al. [8, 15]. Combined with the traditional customs and contemporary national conditions of China, we considered that this may be related to the different labor conditions of different groups, family role positioning and so on. Liu et al. found that SBP increased significantly in the later half of long working hours, indicating that the risk of ICH may be related to intensity and time of working [36, 37]. Female in Farmers and Freelancers mainly take the responsibilities of household chores as a traditional role in the family, including buying groceries and cooking, etc. They may continue perform housework after finishing work in the afternoon [38]. This could explain their early evening peak. Female in Wage-earners may be less traditional about their role in the home, seeing themselves as breadwinners and working like male [39]. They may be consequently less likely to be a homemaker after working hours. This can contribute to the reason for both male and female in Wage-earners exhibited the same peak distribution pattern in the afternoon. We found no difference in the afternoon peak among farmers of different age groups, which may be related to the same working pattern whether they are young or old [40]. However, for Wage-earners 65 years or older, they are generally retired and do not work [41], this may be why they are different from Wage-earners younger than 65 years in the afternoon peak time.

At present, no study has been found on the relationship between circadian variations of ICH onset and working style and living habits of population groups. This study is the first to explore whether working style and living habits have effect on ICH onset time by analyzing circadian variations of ICH onset from different sources of income. Our study demonstrated significant differences in the circadian variations of ICH onset from different sources of income population groups. Not only does this finding provide evidence of working style and living habits can influence circadian variations of ICH onset, but also provides the possibility for further research on how the work-life rhythm affect the circadian variations of ICH onset. In addition, it can help us to provide individualized management strategies for patients with ICH.

Our study has certain limitations apart from the inherent limitation of the retrospective design. First, we only analyzed data on patients admitted to hospitals and did not do a population based study. Therefore, patients who may be missed including those who died before admission and those who did not visit the medical department. These missing patients cannot be grouped accurately because they could belong to any group. However, from experience, we can probably infer that most of them may belong to farmers, the reason may be that farmers’ low socioeconomic status leads to insufficient awareness of disease and lack of economic ability. We believe that this situation may underestimate the incidence of ICH in a specific population, but has little effect on the circadian variation of ICH onset. Population-based studies may better present conclusions that are in line with local populations, but due to the lack of local databases, population-based studies cannot be done at present. The establishment of the corresponding database to make up for these deficiencies is what we expect to be able to complete in the future. In addition, the factors affecting socioeconomic status are very complex and specific in China, so no consensus or criteria for socioeconomic status is available so far. The information about income of patients is not collected routinely by the hospital. Therefore, factors related to socioeconomic status can not be analyzed in deepth in our study. Furthermore, according to the existing data, patients were divided into 3 groups by sources of income: Farmers, Wage-earners, and Freelancers. However, patients in Wage-earners and Freelancers might have some differences in working style due to the different nature of the specific work, especially for freelancers. For example, night shift workers may be classified in the Wage-earners group, and there may also be patients with regular work-life rhythm in Freelancers, therefore, the simple grouping based on the data at hand might not reflect their actual working style. Finally, the living habits of the 3 groups of patients we mentioned was based on the general rules of the local population, but individual differences may exist, such as the habit of napping, the role in the home and the time to get up in the morning. This was an inevitable limitation of our study. The latter two limitations can make the results of our study mainly represented patients in the 3 groups who conformed to general rules, rather than all patients.

## Conclusions

Our study is the first to demonstrate the circadian variations of ICH onset time in patients with different income sources in southwest China’s Chongqing Municipality cohort. These appearances are in accordance with the daily life characteristics of the local people and the routines among the people of different income sources. Although one or more peak hours can be found in ICH onset time in a day, the frequency and distribution pattern of peak hours were closely related to the working style and living habits of people with different income sources. Therefore, we speculate that the work-life rhythm may be a very important factor affecting the onset time of ICH, this may provide the basis for a larger scope of investigation in the future, and provide the possibility for further research on how the work-life rhythm affect the onset time of ICH specific mechanism. Meanwhile, recognition of these particular circadian patterns in ICH is important in planning preventive and control strategies for the sudden, catastrophic cerebrovascular events.

## Data Availability

The datasets used and/or analyzed during the current study are available from the corresponding author upon request.

## Abbreviations

ICH: Intracerebral hemorrhage
NESS-China: National Epidemiology Survey of Stroke in China
CT: Computed tomography
MRI: Magnetic resonance imaging
SAH: Subarachnoid hemorrhages
HTN: Hypertension
SBP: Systolic pressure
DBP: Diastolic blood pressure
PHT: Prehypertension
TC: Total cholesterol
TG: Triglyceride
LDL-C: Low-density lipoprotein cholesterol
DM: Diabetes mellitus
FPG: Fasting blood glucose
RR: Relative risks
CI: Confidence intervals
BP: Blood pressure
